# Development of Optimized Phenomic Predictors for Efficient Plant Breeding Decisions Using Phenomic-Assisted Selection in Soybean

**DOI:** 10.34133/2019/5809404

**Published:** 2019-07-28

**Authors:** Kyle Parmley, Koushik Nagasubramanian, Soumik Sarkar, Baskar Ganapathysubramanian, Asheesh K. Singh

**Affiliations:** ^1^Department of Agronomy, Iowa State University, Ames, IA, USA; ^2^Department of Electrical Engineering, Iowa State University, Ames, IA, USA; ^3^Department of Mechanical Engineering, Iowa State University, Ames, IA, USA

## Abstract

The rate of advancement made in phenomic-assisted breeding methodologies has lagged those of genomic-assisted techniques, which is now a critical component of mainstream cultivar development pipelines. However, advancements made in phenotyping technologies have empowered plant scientists with affordable high-dimensional datasets to optimize the operational efficiencies of breeding programs. Phenomic and seed yield data was collected across six environments for a panel of 292 soybean accessions with varying genetic improvements. Random forest, a machine learning (ML) algorithm, was used to map complex relationships between phenomic traits and seed yield and prediction performance assessed using two cross-validation (CV) scenarios consistent with breeding challenges. To develop a prescriptive sensor package for future high-throughput phenotyping deployment to meet breeding objectives, feature importance in tandem with a genetic algorithm (GA) technique allowed selection of a subset of phenotypic traits, specifically optimal wavebands. The results illuminated the capability of fusing ML and optimization techniques to identify a suite of in-season phenomic traits that will allow breeding programs to decrease the dependence on resource-intensive end-season phenotyping (e.g., seed yield harvest). While we illustrate with soybean, this study establishes a template for deploying multitrait phenomic prediction that is easily amendable to any crop species and any breeding objective.

## 1. Introduction

Soybean [*Glycine Max* (L.) Merr.] breeding programs have improved the crop genetic potential, while producers have modified their agronomic methods to increase seed yield (SY) [1–5]. While genomic-assisted breeding methods are now more routinely applied in large resource-rich breeding organizations, the development of phenomic-assisted breeding methods is in relative infancy and is amendable for cost-effective deployment [6]. High-throughput phenomics has been proposed as a solution to lessen the throughput capacity, mechanical, and resource limitations that exist in plant breeding programs associated with phenotyping [7]. Studies have shown high correlation between phenomic traits collected using digital sensors and manually collected measurements [8, 9] suggesting phenomic data can be acquired on a wide spatiotemporal scale by leveraging the technological advancements made in sensor technology with ground and aerial-based phenotyping platforms [10]. Empowered with phenomic data that was previously difficult or impossible to collect across an expansive spatiotemporal scale, scientists have begun disentangling the genetic architecture of traits through genomic studies [8, 11, 12], prediction of target trait performance using genomic [13–16], and phenomic prediction strategies [9, 15, 17–20]. However, increasing soybean seed yield and on-farm profitability is the primary objective of soybean breeding programs making seed yield an important trait to target in both cultivar and germplasm breeding efforts utilizing phenomics tools that can lead to reduced environmental and genotype testing.

Research has been conducted across several crop species, including soybean, demonstrating the use of phenomic tools to measure traits such as canopy temperature (CT) [16], canopy area [17], and canopy spectral reflectance [18–21] for seed yield prediction. For a phenomic trait to be a useful predictor of seed yield, it must have the following attributes: (a) high genetic correlation with seed yield indicating that the shared additive genetic variation is captured in the phenomic trait, and (b) must be highly repeatable and heritable [22, 23]. Given the complexity of physiological processes responsible for seed yield [2–5] it is likely that a myriad of phenomic traits are required for accurate seed yield prediction across a wide spatiotemporal scale. Studies including phenomic traits in multivariate genomic selection (GS), designed to leverage the shared genetic correlation between traits, have shown increased prediction accuracy proposing the added advantage of including phenomic traits in evaluating candidate genotypes over using yield alone to measure breeding value [14–16]. However, more information is needed on deploying high-dimensional phenomic information to compare the predictability of phenomic traits simultaneously for use in seed yield prediction since breeding programs rely on identifying elite cultivars through empirical as well as prediction based approaches [24].

Given the throughput capacity of high-throughput phenotyping platforms to collect multiple sensor information simultaneously, plant scientists are often left with a high-dimensional phenomic data cube [25]. The ability to handle large amounts of complex data and to capture complex nonlinear relationships between phenomic predictors and seed yield makes machine learning (ML) a viable mathematical tool [9, 26]. Random forest [27] (RF), an ensemble learning ML method, provides the added benefit of using multiple decision trees to model complex trait relationships and the ability to concurrently gauge feature importance to enable the user to glean insights on how predictions were made. In addition to predicting seed yield, identifying an informative subset of predictors is important to reduce data redundancy, minimize sensor cost, and reduce the computational demand required for processing and analysis [28]. Random forest approaches provide simpler interpretability, although advances in deep learning models include explainability of features used in the models for phenotype and this is a rapidly advancing field [29]. In addition to prediction, optimization routine is needed for efficient phenomics based predictors to minimize cost and temporal requirements of data collection. Genetic algorithm (GA) is an optimization algorithm that has been used to identify informative hyperspectral wavebands for disease classification [9, 26, 28], wheat yield and nitrogen status prediction [30], and corn pollen shed detection [31]. GA is designed to mimic natural evolutionary processes to evaluate the performance (fitness) of a group (population) of predictors (chromosomes) and using selection theory to “breed” a new generation of individuals of each generation using a fitness metric to guide the search process so that only the most elite individuals are recombined until some criteria are met [32]. The premise of GA imparts it the ability to select a subset of hyperspectral wavebands to be concurrently deployed on multisensor payload on aerial based platforms for SY prediction, identification of useful genetic diversity [11, 33, 34] (for a more in-depth review on this subject see [35, 36]), and breeding decisions for population advancement and line selection. While significant strides have been made in the use of the visible and near-infrared spectrum, exploring the extent of the spectrum which is currently collectable remains an elusive target.

This work is motivated by the overall need to explore soybean genetic diversity for SY, development of phenomic predictors of SY across growth and development stages using multiple sensors, and data analytic approaches to glean informative pieces of information from a large dataset. Additionally, there is a need to minimize the cost and dedicated resources required for germplasm breeding efforts and to increase the operational efficiency. Therefore, the objectives of this research were (1) to explore and assess the importance of phenomic traits for SY prediction using a diverse set of 292 soybean accessions, (2) to use machine learning and optimization methods to develop prediction models enabling in-season SY prediction and identify informative subset of hyperspectral wavebands for potential phenomic applications to improve SY, and (3) to test and validate prediction models for multiple trait based SY selection. Since most of the yield prediction studies have relied on vegetation indices and canopy traits (area and temperature), we looked at an integrated approach of optimizing the selection of traits and expanding our search space to include individual wavebands.

We propose a framework that is easily transferable to different crops species and breeding program that is looking to fuse ML-based analytics and optimization tools with high-dimensional phenomics data to develop economical but scalable sensor solutions to be deployed using modern phenotyping platforms. These findings present germplasm breeders with an approach to expand testing capacity while improving the accuracy of yield estimation, critical to efficiently mine genetic diversity and drive genetic gain.

## 2. Materials and Methods

### 2.1. Germplasm

We evaluated 292 diverse soybean accessions from 19 different countries adapted to the maturity group (MG) late I to early III (Table S1). Accessions were sourced from the soybean core collection of the USDA Soybean Germplasm Collection [37] and parents of the Soybean Nested Association Mapping (SoyNAM) panel [38] consisting of 260 and 32 accessions, respectively. These accessions were selected to represent the genetic diversity available to the US soybean breeders and can be classified into three genetic backgrounds (https://www.soybase.org/SoyNAM/): (1) elite, (2) diverse, and (3) plant introduction (PI). Elite cultivars consisted of public breeding lines developed by breeders across the US, diverse lines were developed through crossing elite and PI germplasm, and PI germplasm consisted of publically available lines from the USDA germplasm collection.

### 2.2. Experimental Design

The data included in this study was collected across six locations over two years (2016 and 2017 growing seasons) (Table S2), and these environment-year combinations are henceforth referred to as environments. To manage spatial variability, an alpha-lattice design was created uniquely at each environment and consisted of two replications with 30 incomplete blocks. Experimental plots were established with a GPS enabled ALMACO (Nevada, IA, USA) cone planter equipped with four row units (76 cm row spacing) and seeded to a length of 4.6 m with 0.9 m alley between plots. Plots were seeded at a rate of 296 K seeds ha^−1^. Once seedling emergence was complete, the number of plants from a 1 m section from a randomly selected portion of the middle two rows was recorded for each plot to determine suboptimal plots for this study. Plots with low seedling emergence determined by observations more than two interquartile ranges below the first quartile were discarded (14 out of 3504 total plots across the six environments).

### 2.3. Phenotypic Data Collection and Processing

In each environment, plots were phenotyped for physiomorphological (phenomic) traits at two time points during the growing season when plots reached the following approximate growth stages: S1: flowering (R1-R2) and S2: pod set (R3-R4) [39]. The inability and impracticality to collect crop growth stage specific data per plot motivated us to collect across the important crop development stages: flowering and pod set. We selected these two approximate growth stages due to the important phenological stages that impact final seed yield as suggested by previous research [2–5]. We ensured that stage specific data were collected as per the two stages by recording per genotype growth stage at each environment (from the first replication) for each set of phenotypic data collected in the study.

During the 2016 growing season, phenomic traits were collected manually using appropriate sensors and equipment. Through a preliminary study (data not presented), it was determined that four to six hours per sensor per environment was required to collect data depending on walking speed and weather conditions. To optimize data collection by minimizing time required for multiple sensor data collection, we constructed a mobile phenotyping platform similar to [15] and deployed during the 2017 growing season. All physiomorphological traits were collected from the middle two rows and data were collected by pushing/pulling the phenotyping buggy up and down passes while simultaneously collecting data from multiple sensors (canopy temperature, canopy area, and canopy spectral reflectance).

Canopy temperature (CT) was measured at four environments using a FLIR VUE Pro R (FLIR, Goleta, CA, USA) infrared camera with a 9 mm lens and 640 × 512 pixel resolution on cloudless days when wind speed was <2.24 m s^−1^. The sensor was suspended 2.0 m above the soil surface in the nadir position and 8-bit JPG image recorded. Plot CT values were extracted using a custom MATLAB (R2017a) script to remove soil background values and mean thermal temperature in degrees Celsius computed for the canopy area remaining after image thresholding. CT data was then corrected for changes in ambient temperature by normalizing by pass which has been shown to increase repeatability [15].

Canopy area (CA) was determined using Canopeo app [40] in MATLAB to estimate fractional green canopy area from RGB images. JPG images were acquired using a Canon T5i camera (Canon U.S.A. Inc., Huntington, NY, USA) with an 18 to 55 mm lens suspended 2.0 m above the soil surface and 20° from nadir. One image was recorded per plot with camera lens zoom fully retracted and camera facing plot so that a landscape image was taken to capture a long transect of the middle two rows. To ensure consistent image quality, images were collected in automatic mode (Program AE) to automatically control both aperture and shutter speeds to maintain consistent exposure value.

Canopy spectral reflectance was measured using a FieldSpec® 4 Hi-Res (ASD Inc., Boulder, CO, USA) spectroradiometer which measures 2150 spectral wavebands from 350 to 2500 nm. Data was collected by positioning the fiber-optic cable 1 m above the canopy in the nadir position and two reflectance measurements were recorded from each of the middle two rows on cloudless days ±2 h of solar noon and calibrated every 20 minutes during data collection by normalizing to a white reference panel (Specralon®, Labsphere Inc., North Dutton, NH, USA).

We processed the data as follows:

Data Processing Step 1: calculated average reflectance for each plot by averaging the two observations.

Data Processing Step 2: computed repeatability for individual wavebands across all locations using the following equation [24]:

(1) $$\begin{array}{c} H^{2}=\frac{\sigma_{g}^{2}}{\sigma_{g}^{2}+\sigma_{GxE}^{2}/e+\sigma_{\varepsilon }^{2}/re} \end{array}$$

Where *σ*_*g*_^2^ is the genotypic variance, *σ*_*GxE*_^2^ is the variance attributed to genotype environment interaction, *σ*_*ε*_^2^ is the residual variance, *r* is the number of replications, and *e* is the number of environments.

Data Processing Step 3: removed wavebands with *H*^2^ < 0.3.

Data Processing Step 4: calculated vegetative indices (VI) previously characterized to be associated with different physiological traits (Table S3).

Data Processing Step 5: computed the mean reflectance across blocks of 10 nm regions (R) across the 1780 wavebands to produce 178 averaged wavebands. We chose to average every 10 nm to reduce multicollinearity between adjacent wavebands and to identify spectral regions with resolution consistent with customizable miniaturized multispectral cameras currently publicly available for research and breeding applications.

Seed yield (SY, kg ha^−1^) was measured from the middle two rows of four row plot by machine harvest using ALMACO SPC20 combine after plots had reached physiological maturity (R8). Seed moisture was measured of harvested plots to adjust plot SY values to 13% moisture. Preharvest shattering was scored for each plot on 1 (no shattering) and 5 (more than 50% of plants had more than 50% of seed loss) scale and yield observations with preharvest shattering score of ≥4 were removed from further analysis (27 out of 3504 total plots across the six environments).

### 2.4. Statistical Model

A mixed linear model was fit to the alpha-lattice design to test model effects and obtain genotypic best linear unbiased predictions (BLUPs) of studied traits using the R package lme4. A mixed linear model was fit with the form:

(2) $$\begin{array}{c} y_{ijkl}=\mu +E_{i}+R_{j}+B_{k\left(j\right)}+G_{l}+E\times G_{il}+\varepsilon_{ijkl} \end{array}$$

where *y* is a vector of observed phenotypes, *μ* is the grand mean, *E*_*i*_ is the effect of the *i*th environment, *R*_*j*_ is the effect of the *j*th replicate, *B*_*k*(*j*)_ is the effect of the *k*th incomplete block nested within the *j*th replicate, *G*_*l*_ is the effect of the *l*th genotype, *E* × *G*_*il*_ is the effect of G x E, and *ε*_*ijkl*_ is the residual error and is assumed to be normally and independently distributed, with mean zero and variance *σ*^2^. Assumptions of ANOVA were tested using Shapiro Wilk test and Bartlett's test using base functions in R. Residuals were normally distributed with homogenous variance. To identify inconsistencies in the data, outliers were removed by calculating studentized residuals for each observation of each trait and outliers excluded from the analysis with values ±3.

Analysis of variance (ANOVA) for seed yield was conducted to evaluate the effect of genotype, termed as fixed, and all remaining termed as random using a mixed linear model with the same as that for (2). Additionally, a two-way ANOVA Dunnett's test was used to compare PI and diverse accessions with elite genotypes as the control and adjusted P-values computed for comparison between each genotype and the control (elite genotypes). Accessions with statistically similar seed yield were defined as P > 0.05.

To deal with missing data at some locations and unbalanced sample size of phenomic information among accessions due to weather or logistical constraints during phenotyping (Table S4), genotype BLUPs were computed using two methods (also see Cross-Validation Section below):

Method 1: from four out of six environments, by-environment BLUPs, were computed as they had complete datasets.

Method 2: across-environment BLUPs were computed for all six environments.

These preprocessing steps of BLUP computation were motivated with the intention to compare phenomic prediction model accuracy when a complete training set is assembled across all environments versus a scenario where environments have sparse phenomic information. Both these scenarios are endemic to germplasm and cultivar development programs conducting multiple environment testing. Method 1 BLUPs were computed by removing all terms associated with environment, while Method 2 BLUPs were computed using (2) with all terms considered random.

### 2.5. Genetic Correlation and SNP-Based Heritability

Genetic correlations (*r*_*g*_) between seed yield and phenomic traits were computed using multivariate mixed models [13]. SNP-based heritability (*h*_*SNP*_^2^) [41] was calculated using a mixed linear model with the form:

(3) $$\begin{array}{c} y=\mu +Zu+E \end{array}$$

Where *y* is a vector of BLUP phenotypic values computed from method 2 for the trait of interest, *μ* is a scalar intercept, **Z** is an incidence matrix for the random genotype term, *u* is a vector of random effects corresponding to genotypes [*g* ~ (0, **A***σ*_*u*_^2^)][*g* ~ 0, *Aσg*2], where A is the additive genomic relationship matrix [42], and *ℰ* is a vector of residuals. Genotypic data for all 292 genotypes was obtained from the publicly available Illumina Infinium SoySNP50K BeadChip database (https://soybase.org/snps/). Single nucleotide polymorphism (SNP) markers with missing rate >10% were removed from the analyses and the remaining missing SNPs imputed using BEAGLE version 3.3.1 with default settings in synbreed package [43]. After imputation, SNPs with minor allele frequency (MAF) <5% were removed leaving 35,512 SNPs. Unlike conventional estimates of heritability, A is used to calculate marker-based genetic variance (*σ*_*g*_^2^) associated with genotypes and *h*_*SNP*_^2^ computed using:

(4) $$\begin{array}{c} h_{SNP}^{2}=\frac{\sigma_{g}^{2}}{\left(\sigma_{g}^{2}+\sigma_{e}^{2}\right)} \end{array}$$

where *σ*_*e*_^2^ is the residual variance (for a more in-depth review see [13, 42, 44]). The R package sommer [45] was used to compute the A matrix, genetic correlation, and *h*_*SNP*_^2^ using the built-in pin function and standard error estimates were computed simultaneously.

### 2.6. Phenomic Prediction Pipeline

In this study, we developed an analytical pipeline using RF algorithm for prediction of SY (response variable) using phenomic traits (predictor variables). Predictive ability of phenomic traits for SY prediction was determined by partitioning predictor traits into three cohorts: (1) canopy (CA and CT), (2) VI, and (3) wavebands. For each cohort, predictor variables were independent factors. Models were trained using (a) canopy alone, (b) VI alone, (c) canopy and VI together, and (4) wavebands alone (see Data Processing Step 5 above). Essentially, sensor combinations that can be easily deployed onto payloads were the key driver in exploring prediction performance for these combinations of sensors. The caret package [46] implemented in R was used for model training and hyperparameters tuned using the tunelength function. To gauge model performance during training, repeated (n=5) 10-fold cross-validation was used and the coefficient of determination (R^2^) and root mean square error (RMSE) for out-of-bag (OOB) samples are reported. Predictions were then projected onto an independent dataset (see Cross Validation section below) not included in model training and consisting of only phenomic traits. Variable importance was computed using the varImp function and mean importance is reported.

### 2.7. Cross-Validation (CV)

**Figure 1:**
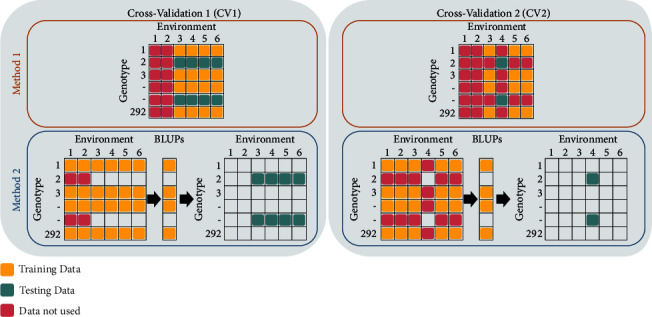
Cross-validation scenarios (CV1 and CV2) and preprocessing methods (Methods 1 and 2) used to assess phenomic prediction model performance. Method 1 and Method 2 differ in BLUP computation, while CV1 and 2 depict two plant breeding scenarios for prediction in multienvironment tests. These CV scenarios represent a combination of different preprocessing (to handle missing data) methods and prediction challenges native to plant breeding practices. In Method 1, for both CV scenarios, individual environment BLUPs are computed and subsequently used in model training and testing the model. In Method 2, combined environment BLUPs are computed and subsequently used in training the model, while individual environment BLUPs are used in testing the model.

To evaluate model performance, we used two cross-validation (CV) scenarios to emulate phenomic prediction in plant breeding programs (Figure 1):

CV1: from all environments, 80% of accessions (n=234) were included in model training set and 20% (n=58) were kept in the testing set.

CV2: this was used for per environment prediction cross-validation and the four environments with complete datasets were included. For each of these four environments, 80% of accessions from the other three environments were used for model training, while 20% of accession for that environment was used for testing; i.e., for Environment#2, model training was done on 80% of random accessions from Environments# 1, 3, and 4, and testing was done on 20% of remaining accession from Environment#2. For CV1 and CV2, the training and testing procedures were repeated 10 times and the mean accuracy for each CV-Method combination is reported. Training and testing sets were compiled for each CV iteration and training data used to parameterize model and prediction made onto the test set following model training.

Two preprocessing methods were used to parameterize RF prediction models (see Statistical Model section), and we then tested two CV scenarios to emulate prediction challenges faced by breeders in field trials with unbalanced data. From a practical application viewpoint, the CV1 strategy is a scenario where phenomic data is collected on all genotypes while yield is collected on a subset of lines and breeders may wish to estimate the rank performance of untested genotypes not phenotyped for yield but with available physiological trait data. The CV2 strategy is deployable where breeders are interested in predicting rank performance of untested accessions (no seed yield data) and untested environments (unseen environment) with no seed yield but with phenomic traits. The CV2 strategy is an improvement to leave-one-environment-out [47] situation as we excluded test genotypes from model training.

Model prediction accuracy is reported using Spearman rank correlation coefficient between observed values and predicted values of the test set computed by recording the mean values across all 10 training-testing iterations and all folds of CV. Cross-validation schemes were developed in R using in-house script.

### 2.8. Predictor Optimization

To identify spectral reflectance wavebands and validate previous findings, we used a genetic algorithm (GA) optimization approach with RF-based predictor as the underlying function evaluator to identify a subset of wavebands capable of being deployed using a multispectral camera. The objective was to identify four wavebands common across the two growth stages (S1 and S2) that maximized seed yield rank correlation while deploying one multispectral camera; therefore our search space spanned the set of 356 wavebands (178 wavebands per growth stage) while ultimately picking the four most optimal wavebands. We chose to identify four wavebands as this is consistent with the current offering of third-party vendors providing customizable cameras that can be used as a selection tool for phenomic-assisted breeding selection processes. Details of the GA process are outlined in Table S5. As GA is a computationally intensive process and prior results showed higher prediction accuracy using Method 1 BLUPs, we limited future analyses to this subset and therefore only Method 1 results are presented. Furthermore, the GA approach was not used in Method 2 (for developing a regression model) due to insufficient dataset size. Using the same training and testing data in the aforementioned phenomic prediction section and once terminal conditions were met, a RF model was retrained and prediction performance assessed by predicting seed yield on the complete testing set using the four selected wavebands and Spearman rank correlation was computed. To supplement wavebands, we selected the VI with the highest *r*_*g*_ in the respective CV scenario and canopy (temperature and area) traits for each CV scenario. In addition to reporting Spearman rank correlation for the test set, we measured breeding success outcome given a hypothetical selection intensity of 20% through a confusion matrix: true positive (TP), true negative (TN), false positive (FP), and false negative (FN). From these values, classification metrics relevant to plant breeding were computed from the confusion matrix output:

(5) $$\begin{array}{c} BalancedAccuracy\left(BAC\right)=\frac{TP/\left(TP+FN\right)}{TN/\left(TN+FP\right)} \end{array}$$

(6) $$\begin{array}{c} FScore\left(FS\right)=\frac{2TP}{\left(2TP+FP+FN\right)} \end{array}$$

(7) $$\begin{array}{c} Specificty\left(SPE\right)=\frac{TN}{\left(TN+FP\right)} \end{array}$$

Spearman rank correlation and confusion matrix results are reported from a study of the effect of training population size using variable training set size: 80% (234 genotypes), 60% (175 genotypes), 40% (117 genotypes), and 20% (58 genotypes) for the optimized RF prediction model in the two CV procedures. Mean predictive performance was assessed for each training population size.

## 3. Results

### 3.1. Seed Yield Performance

**Figure 2:**
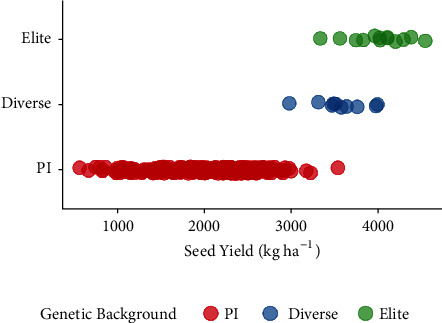
Machine harvested seed yield (kg ha^−1^) of 292 genotypes grouped as elite (n=13), diverse (n=10), and PI (n=269). Tests were grown across two years in six environments across central Iowa. Seed yield was computed from combined environment BLUP.

A significant effect of genotype, environment, and their interaction was observed (Table S6). Mean SY of 2113 kg ha^−1^ was observed across the 292 accessions with elite germplasm (4008 kg ha^−1^) having superior SY followed by diverse (3570 kg ha^−1^) and PI (1968 kg ha^−1^). The extent of seed yield performance was extensive: 566-3537 kg ha^−1^ within the PI cohort, 2979-3991 kg ha^−1^ within diverse accessions, and 3335-4542 kg ha^−1^ within the elite accessions. Three diverse accessions were not significantly different compared to the mean performance of the elite accessions. While the most extensive trait variation was observed for PI, there was an overlap in performance of the three groups (Figure 2). PI597482 (from South Korea) had the highest SY (3537 kg ha^−1^) within the cohort.

### 3.2. Genetic Correlation and SNP-Based Heritability

**Figure 3:**
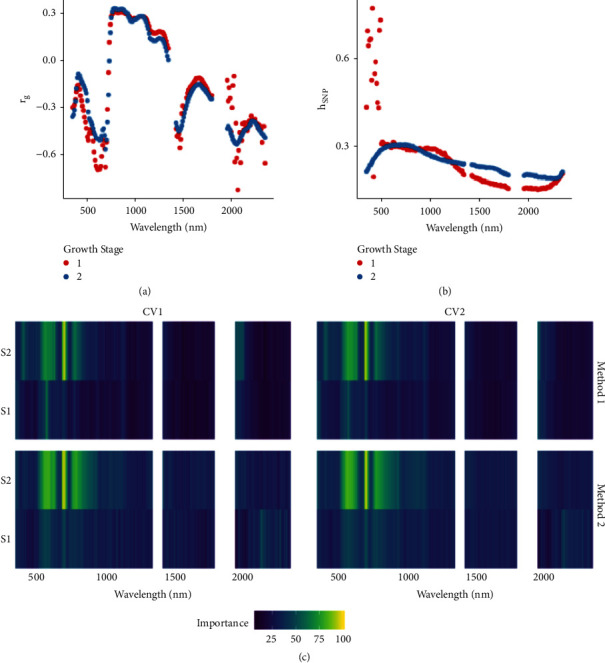
Analysis of hyperspectral canopy reflectance wavebands (average reflectance per 10 nm) and relationship with seed yield using 292 soybean genotypes grown in six environments (replication per environment = 2). (a) Genetic correlation (*r*_*g*_) between seed yield and waveband, (b) SNP-based heritability (*h*_*SNP*_^2^) across waveband, and (c) feature importance for predictor variables (i.e., waveband) for SY estimation using the random forest algorithm. Hyperspectral canopy reflectance data were collected in six environments across central Iowa by recording two measurements by positioning the sensor 1 m above the canopy in the nadir position.

The genetic correlation (*r*_*g*_) among SY and independent variables (canopy traits, VI, and wavebands) in both growth stages had a large range: -0.80 to 0.60 in S1 (flowering) and -0.75 to 0.59 in S2 (pod set) (Table S7, Figure 3(a)). Among canopy traits and VI, Vogelmann Red Edge Index 2 (VREI2) had the strongest *r*_*g*_ with seed yield of -0.77 and -0.75 in S1 and S2, respectively. Other VIs identified with strong *r*_*g*_ were Normalized Water Index (NWI) (S1: -0.58, S2: -0.59), Ratio Analysis of Reflectance Spectra Chlorophyll b (RARSb) (S1: 0.59, S2: 0.50), and Ratio Analysis of Reflectance Spectra Chlorophyll c (RARSc) (S1: 0.60, S2: 0.43). The *r*_*g*_ of SY canopy traits were 0.33 (S1) and 0.25 (S2) with CA, and -0.44 (S2) with CT. VI NMDI exhibited a strong dependency of growth stage on *r*_*g*_ resulting in a 180% change from S1 (0.03) compared to S2 (0.59). The *r*_*g*_ between canopy spectral reflectance wavebands and SY was highly variable (-0.82 to 0.32) across the electromagnetic spectrum but followed a consistent trend for both collected growth stages (Figure 3(a)). Two regions across the electromagnetic spectrum were identified with strong *r*_*g*_ in the visible to near-infrared region (700-850 nm) and in the shortwave infrared regions (2030-2119nm). Strong *r*_*g*_ between SY and waveband reflectance was observed with 705 nm waveband (average wavelength in nm) across both growth stages (S1: −0.67, S2: −0.56) while the maximum absolute *r*_*g*_ was observed for 2065 nm (S1: −0.82, S2: −0.52).

Consistent with *r*_*g*_, SNP-based heritability (*h*_*SNP*_^2^) analysis revealed a wide range from 0.07 to 0.77 in S1 and 0.19 to 0.73 in S2 for phenomic traits (Table S7, Figure 3(b)). SY *h*_*SNP*_^2^ was 0.32. VIs had higher *h*_*SNP*_^2^ in S2 (0.54) when compared to S1 (0.30). VI NDVI had the highest *h*_*SNP*_^2^ in S2 (0.51) while VREI2 had the highest *h*_*SNP*_^2^ across both growth stages (S1: 0.51, S2: 0.65). The *h*_*SNP*_^2^ for CA was higher in S1 (0.50) compared to S2 (0.38) while CT, measured at S2, was 0.29. Waveband *h*_*SNP*_^2^ ranged from 0.15 to 0.77 in S1 and 0.19 to 0.31 in S2 and revealed a similar decreasing trend across the spectrum and maximum *h*_*SNP*_^2^ (0.77) was observed in S1 in the visible region (Figure 3(b)).

### 3.3. Phenomic-Enabled Yield Prediction

**Figure 4:**
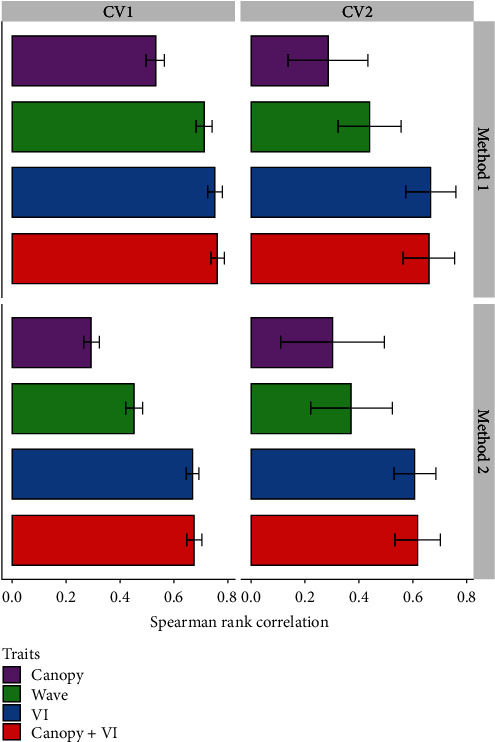
Spearman rank correlation obtained after random forest model prediction (seed yield = dependent variable) performance of predictors trained with remotely sensed phenomic traits (canopy traits, waveband, vegetation indices, and combination) in 292 soybean genotypes grown at six environments and data collected at two growth stages in each environment. Error bars represent standard deviation around the mean.

Overall, we observed the following trends: (1) phenomic data collected at two growth stages during the growing season was predictive of SY rank at maturity, (2) the use of by-environment BLUPs had improved prediction accuracy compared to using across-environment BLUPs for predicting seed yield, (3) RF model had improved prediction accuracy when training data was included from the same environment in which the test genotypes were evaluated, and (4) a wide range in prediction accuracy was observed among predictor cohorts demonstrating the need for identification of the best predictors to optimize sensor deployment (Figure 4).

Higher rank correlation in CV1 was observed when compared to CV2, and higher rank correlation in Method 1 was observed in comparison to Method 2. The four-way classification of Method (1 and 2) and CV (1 and 2) showed that there was an increase in rank correlation from canopy (0.35) < waveband (0.49) < VI (0.67) < canopy + VI (0.68) (Figure 4). Canopy rank correlation increased by 62% with the addition of VIs (canopy + VI) and minimal change was observed between canopy + VI and VI (<1% difference). Method 1 (training set using by-environment BLUPs) had 18% higher rank correlation than Method 2 (across-environment BLUPs). CV1 (unknown accessions) had 22% higher rank correlation when compared to CV2 (unknown accession in unknown environment). Maximum rank correlation was observed for canopy + VI in Method 1 (0.76) and Method 2 (0.68). Moderate rank correlation (0.49) was observed using 178 raw reflectance wavebands per growth stage. When wavebands were considered, higher rank correlation was observed in Method 1 compared to Method 2 and CV1 compared to CV2 (34% higher in each).

Variable importance analysis revealed CA and VREI2 were most important for models trained using canopy and VIs, respectively (Table S8). Wavebands in the visible to near-infrared region were most important overall and were consistent across CV scenarios and preprocessing methods (Figure 3(c)). Wavebands collected at S2 growth stage had higher importance than those collected in S1. Waveband 715 nm was identified as the most important across all growth stages. In Method 1, wavebands in the shortwave infrared region were also important to model prediction.

### 3.4. Phenomic Predictor Optimization and Its Application

**Figure 5:**
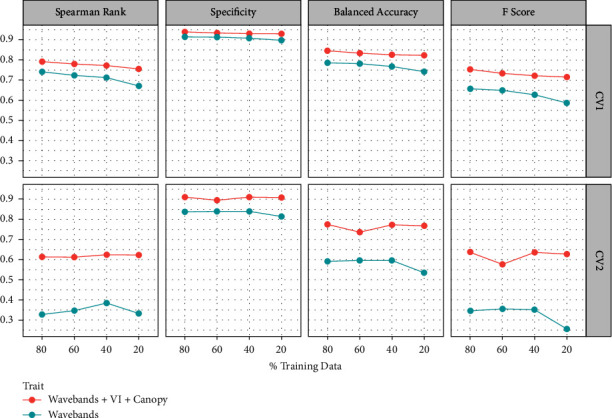
Spearman rank correlation and classification metrics (specificity=SPE, balanced accuracy=BAC, F score=FS) of random forest model test prediction using only optimized wavebands (blue line) and selected canopy traits (red line). Applicability of using phenomic prediction in plant breeding operations was tested using four training/testing splits (80/20, 60/40, 40/60, 20/80) and performance metrics were computed for each split. Seed yield and phenomic predictor trait data were collected from 292 genotypes grown in six environments and data collected at two growth stages in each environment.

The majority of selected wavebands GA step were in the visible region: 405 nm, 435 nm, 705 nm, 715 nm, two in near-infrared region: 795 nm, 815 nm, and one in the shortwave infrared region: 2255 nm. The most predictive bands for CV 1 were 435 nm, 705 nm, 815 nm, 2255 nm, while for CV2 were 405 nm, 705 nm, 715 nm, 795 nm. Based on our results on *r*_*g*_ and feature importance analysis, and the ease of deployment of different sensors, VREI2, CA, and CT were chosen along with most predictive wavebands for testing their SY prediction performance (Figure 5). Prediction performance (Spearman correlation) of CV1 and CV2 was 0.74 and 0.33, respectively. A slight increase in rank performance was noticed in CV1 when GA generated bands were used (rank correlation increased by 0.03) and a slight decrease observed in CV2 (rank correlation decreased by 0.11). High specificity (SPE) was observed among all models ranging from 0.81 to 0.94 and was slightly higher for models trained in CV1 (0.92) compared to CV2 (0.87). Similarly, moderate to high F score (FS) and balanced accuracy (BAC) was observed for all CV-model combinations with higher values for CV1 compared to CV2.

As the amount of training data was reduced from 80% to 20%, models including wavebands + VI + canopy have consistently higher performance for rank correlation (28% higher) and classification metrics (18% higher). Spearman rank correlation decreased slightly for both models trained in CV1 (waveband + VI + canopy: 0.04 reduction, wavebands alone: 0.07 reduction) when comparing prediction performance trained using 80% of the data when compared to using just 20%. Minimal decrease in SPE was observed with just an average decrease in performance of 0.01 when using the minimum amount of training data, compared to using 80%. The largest change was observed for BAC and FS with an average reduction of 0.03 and 0.06, respectively. The largest change was observed when wavebands alone were used for model training in CV2 resulting in a 10% and 26% reduction in BAC and FC, respectively.

## 4. Discussion

Breeders and geneticists aim to utilize previously unused genetic accessions in cultivar development, and phenomic-assisted breeding approaches have the potential to enhance the integration of genetic diversity in most mainstream programs [36]. Phenomic-assisted approaches can allow breeders to manipulate the genetic gain equation, particularly genetic variation and selection intensity. For improving SY using diverse accession, as a first step, there is a need to establish the relationship between phenomic traits with SY using high-throughput phenotyping techniques and advanced data analytics including machine learning [9]. These approaches need to work in conjunction with in-season SY prediction, but more importantly performance ranking that is the crux of trait selection in plant breeding programs.

We identified a cohort of PI accessions with high yield, further demonstrating the wealth of genetic diversity available to soybean breeders in the germplasm collection. These results are consistent with a broader body of research demonstrating the utility of germplasm collection for modern breeding efforts for biotic [48–50] and abiotic [51–53] resistance and performance traits [33, 54–56]. The presence of genetic variation for SY makes this panel of 292 accessions relevant for study objectives as it covers a broader range of performance and background.

### 4.1. Phenomic-Enabled Yield Prediction

Moderate to high *h*_*SNP*_^2^ for all traits suggest that phenomic trait measurements are repeatable making them useful in plant breeding pipelines. Spearman-rank correlation coefficient was used to assess model test performance as plant breeders are generally focused on correctly identifying top performers in early to mid-stages of testing pipeline instead of predicting actual SY [23]. The identification of best predictors for phenomic-enabled rank correlation is important to maximize prediction accuracy thereby maximizing the detection of useful germplasm for use in cultivar development and also for selection of pure lines in breeding families from multienvironment tests.

Plant breeders often rely on multienvironment trials to evaluate cultivar performance in a target environment, quantify GxE interaction, and/or determine cultivar stability [57]. On average, we observed 18% higher prediction accuracy when training data consisted of BLUPs generated on a by-environment basis when compared to using across-environment BLUPs. The use of mixed models for computing BLUPs is a staple in plant breeding statistical analyses and a main feature of the method is its ability to handle missing or unbalanced data, a common occurrence in multienvironment trials (MET) [24]. When complete data is generated in all environments, a single stage analysis [58] is preferred to preserve the environmental effect in the data. Nonetheless, assembling complete data in all environments is often not the case and therefore relying on the properties of the BLUP method is necessary to remove the experimental design effect from the estimates and simultaneously taking advantage of the amendable variance-covariance structure for genotype-by-environment (GxE) interactions [24]. Additionally, there is a setting off of prediction based selection and resource optimization which are popularizing experimental designs such as partial replication design in plant breeding programs [59]. The RF model accuracy was 22% higher when prediction was made in locations included in model training. We observed that RF models had higher prediction accuracy when by-environment BLUPs were used in model training; moderate accuracy levels were still attainable even when environments with sparse data were included in model training indicating the reaction norm across locations for phenomic trait relationships with SY was somewhat consistent in each environment. These findings demonstrate the impact that environment has on genotype performance and is evidence of the importance for having training data in environments reflective of the target breeding area.

The variation in prediction accuracy among predictor cohorts across the two preprocessing methods and two CV scenarios suggests that multiple trait information can help gain operational efficiencies. We observed moderate *r*_*g*_ (S1: 0.33, S2: 0.25) between CA and SY is lower than previous studies [17] although the trait genetic correlation was observed in a biparental population. CT exhibited negative *r*_*g*_ (-0.44) with yield and shows congruence with previous studies [16, 53, 54]. We observed dissimilarity between some phenomic traits with previously reported [5, 17] canopy traits (CA and CT) produced only modest prediction accuracies. We observed a significant improvement when VIs were included in the model. Among VIs, VREI2 had the largest *r*_*g*_ in magnitude (S1: -0.77, S2: -0.75) and is associated with chlorophyll concentration, water content, and canopy leaf area [60] and lends support to the utility of VREI2 as a yield predictor VI [11] since gain in genetic yield potential in soybean has been associated with an increase in canopy chlorophyll concentration [2, 4, 61]. Moreover, we report moderate to high *r*_*g*_ in the shortwave infrared region, a region associated with plant water potential [62]. Research in wheat [63, 64] and corn [18, 65] using VIs associated with plant water content in shortwave infrared waveband regions has shown good correlation with yield; however, similar reports in soybean are lacking warranting additional investigation to associate shortwave infrared canopy spectral reflectance with yield especially to develop water deficit tolerant cultivars. Since majority of 292 accessions belonged to PI accessions, it was not surprising to see the value of chlorophyll based VI as an important predictor. For cultivar development programs, the role of chlorophyll based VI needs to be investigated prior to implementation in breeding selection.

The combination of high repeatability and genetic correlation makes phenomic traits useful in indirect selection for SY. Additionally, our results reveal that canopy spectral reflectance wavebands can be useful for yield prediction as reported by [19] and suggest that informative wavebands may be identified to design a multispectral camera for use in extremely high-throughput aerial-based phenotyping. Phenomic prediction has the potential to disrupt conventional breeding testing pipelines by integrating information on important biological processes across a spatiotemporal scale to enable in-season yield assessment and optimizing plant breeding operation efficiencies [7] and requires an interdisciplinary approach.

### 4.2. Phenomic Predictor Optimization and Its Application

Optimizing the deployment of phenomic sensors specific to the breeding target is an important objective to maximize prediction accuracy while reducing the operational costs associated with data collection. However, there remains a gap in the current understanding of the utility of a multisensor approach for SY prediction to identify the optimal sensors for use in soybean germplasm breeding efforts.

Our results show the utility of canopy spectral reflectance for use in SY rank prediction using wavebands and VIs and are consistent with previous research findings made in soybean [11, 20, 21, 66] and other crop species [19, 30, 67, 68] for trait prediction; however, the utility of waveband reflectance as a predictor has not been extensively studied. Therefore, we chose to identify four wavebands which can allow the design of multispectral camera consistent with the current options available from industry providers offering customizable waveband selection of multispectral cameras. To do this, a genetic algorithm (GA) approach was used to identify wavebands for SY prediction. GA has been used for a wide variety of objectives in agriculture for variable and waveband selection [28, 32, 69, 70] but limited work has been done for use in prediction of SY. Research has shown good prediction performance of models using all measured wavebands in wheat [19], but our results suggest that a subset can be used to achieve comparable prediction performance (Figure 4). This finding is likely due in part to the multicollinearity associated with neighboring wavebands allowing a subset of wavebands to capture the variation in entire electromagnetic spectrum [30, 71]. While previously the waveband regions we report have been shown to be correlated in the visible and near-infrared regions of the electromagnetic spectrum [11, 21, 66], GA methodology enabled us to identify specific wavebands for SY prediction. The observation of wavebands in the shortwave infrared region important for yield prediction warrants additional research to explore this portion of the electromagnetic spectrum along with the need for future research to determine the physiological basis of wavebands and their prediction. The next step in SY prediction deployment in a breeding pipeline is the motivation to increase model prediction accuracy by combining multiple sensors as well as resolving challenges on spectral reconstruction from images [72, 73]. While selected hyperspectral wavebands can be deployed on high-throughput phenotyping platforms using multispectral cameras, a multisensor approach needs to be tested to determine if it can maximize model prediction accuracy.

Past studies have established the use of single sensor-based prediction methods in plant breeding activities [14, 16, 18–20, 65, 74, 75] and multisensor based prediction in wheat [15]; however, there is little information on the use of multisensor based prediction in soybean. Thus, we selected VI VREI2, CA, and CT as these traits can be collected in tandem with a multispectral camera and have demonstrated strong *r*_*g*_ and/or moderate to high feature importance to SY. Thus, we observed maximum prediction accuracy when a multisensor based model was used for prediction of SY. Thus, we propose this framework to deploy a multisensor based approach by relying on feature importance parameters and optimization procedures to maximize target trait prediction accuracy.

To determine the value of these approaches for use in plant breeding operations we varied the training/testing split and used a hypothetic selection intensity of 20%; both operational decisions breeding programs attempt to optimize [23]. These findings indicate that, when training data is collected from the same environments in which testing is done, phenomic prediction can be effective to correctly rank genotypes for SY. Moreover, high SPE (ability of the model to correctly identify accessions that did not meet our imposed selection criteria according to ground-truth yield data) was achieved regardless of both the CV scenario and the amount of training data used. While only slightly lower performance was observed for other classification metrics (BAC and FS), our results continue to suggest the efficacy of such phenomic prediction methodologies for breeding decision making. We anticipate that phenotyping and data analytics operability difficulties may need to be resolved for multiple sensor payload and balancing with area coverage of aerial systems and real-time of quick-turn around analytics and remain an area of research interest.

In order for phenomic traits to be informative predictors of target traits high genetic correlation among target-predictor traits (*r*_*g*_) and high predictor trait heritability (*h*_*SNP*_^2^) [23] are needed. Continued work is needed to provide insight into the attribution of phenomic traits for phenomic predictive ability and establishing the biological and physiological association between target traits with predictor traits. Future research is warranted to determine program and trait specific predictors, and such research requires larger datasets. As the hardware and analytics pipelines advance through continued improvement in high-throughput phenotyping, larger datasets will be achievable.

As a selection tool, our approach permits SY rank prediction and will allow the evaluation of specific trait efficiencies to identify useful germplasm on a per-trait basis and design future crossing combinations that assemble desirable traits together. This is a keystone concept in the process of physiological trait based breeding [76, 77]. Overall, our findings suggest that a customized suite of phenomic sensors can advance germplasm and cultivar breeding efforts while reducing the cost and resource requirements and advance the integration of phenomic-assisted breeding approaches. The approach we propose can be utilized in breeding programs to identify informative waveband combinations tailored to the specific breeding objective for the design of customizable multispectral sensors. Our approach can be utilized as standalone but does not preclude the use of wavebands that have been traditionally used to compute various VIs.

While GS and other modern tools will remain an attractive arsenal in a breeder toolbox, the cost of GS assisted breeding can be out of reach for majority of programs in minor crops and in non-GM crops [7] and therefore cost affordable phenomic-assisted breeding approaches present exciting avenues for trait improvement including a multiobjective optimization scenario [78].

## Data Availability

Data are available upon request.

## Supplementary Materials

Supplementary Materials Table S1: description of accessions, country of origin, and genetic background included in this study. 292 accessions were selected from the USDA Soybean Core Collection from MGI-III. Table S2: description of testing environment locations, planting date, seed yield (SY) performance, and climactic summary statistics. Soybean accessions were phenotyped in these environments for use in downstream phenomic prediction. Table S3: description of vegetation indices (VI) computed from canopy hyperspectral reflectance. Observations consisted of two measurements recorded within 2 hours of solar noon and mean reflectance averaged. VIs were used alongside other phenomic information for in-season seed yield prediction [79–85]. Table S4: description of phenotypic traits and instruments used for phenotypic characterization of a diverse panel of soybean evaluated in six environments. Table S5: details of genetic algorithm (GA) procedure used for selection of hyperspectral wavebands for identifying the most informative wavebands to allow intelligent design of a miniaturized hyperspectral camera for deployment on high-throughput phenotyping platforms. Table S6: ANOVA results of fixed effects for mixed linear model where seed yield (SY) was the response variable. SY was collected from 292 genotypes grown in six environments across central Iowa and measured by combine harvest. Table S7: genetic correlation (*r*_*g*_) and SNP-based heritability of phenomic traits and seed yield and phenomic trait, respectively. Phenomic information was collected from 292 diverse soybean accessions grown in six environments across central Iowa and data collected during the growing seasons at two approximate growth stages. Table S8: phenomic traits feature importance computed from random forest model using two cross-validation scenarios while seed yield was used as the response variables. Phenomic traits were collected at two approximate growth stages and used to predict seed yield during the growing season to enable in-season selection. Feature importance was used to select the most informative vegetation indices and to identify other useful predictors of seed yield. Table S9: Spearman rank correlation obtained after random forest model prediction (seed yield = dependent variable) performance of predictors trained with remotely sensed phenomic traits (canopy traits, waveband, vegetation indices, and combination) in 292 soybean genotypes grown at six environments and data collected at two growth stages in each environment. Tabular data correspond to Figure 4. Table S10: Spearman rank correlation and classification metrics of random forest model test prediction using only optimized wavebands and selected canopy traits. Applicability of using phenomic prediction in plant breeding operations was tested using four training/testing splits (80/20, 60/40, 40/60, and 20/80) and performance metrics were computed for each split. Seed yield and phenomic predictor trait data were collected from 292 genotypes grown in six environments and data collected at two growth stages in each environment. Tabular data correspond to Figure 5.

## References

[1] Suhre J. J., Weidenbenner N. H., Rowntree S. C. (2014). Soybean yield partitioning changes revealed by genetic gain and seeding rate interactions. *Agronomy Journal*.

[2] Specht J., Hume D., Kumudini S. (1999). Soybean yield potential—a genetic and physiological perspective. *Crop Science*.

[3] Koester R. P., Skoneczka J. A., Cary T. R., Diers B. W., Ainsworth E. A. (2014). Historical gains in soybean (Glycine max Merr.) seed yield are driven by linear increases in light interception, energy conversion, and partitioning efficiencies. *Journal of Experimental Botany*.

[4] Jin J., Liu X., Wang G. (2010). Agronomic and physiological contributions to the yield improvement of soybean cultivars released from 1950 to 2006 in Northeast China. *Field Crops Research*.

[5] Keep N., Schapaugh W., Prasad P., Boyer J. (2016). Changes in physiological traits in soybean with breeding advancements. *Crop Science*.

[6] Furbank R. T., Tester M. (2011). Phenomics - technologies to relieve the phenotyping bottleneck. *Trends in Plant Science*.

[7] Tardieu F., Cabrera-Bosquet L., Pridmore T., Bennett M. (2017). Plant phenomics, from sensors to knowledge. *Current Biology*.

[8] Zhang J., Naik H. S., Assefa T. (2017). Computer vision and machine learning for robust phenotyping in genome-wide studies. *Scientific Reports*.

[9] Singh A., Ganapathysubramanian B., Singh A. K., Sarkar S. (2016). Machine learning for high-throughput stress phenotyping in plants. *Trends in Plant Science*.

[10] Gao T., Emadi H., Saha H. (2018). A novel multirobot system for plant phenotyping. *Robotics*.

[11] Dhanapal A. P., Ray J. D., Singh S. K. (2016). Genome-wide association mapping of soybean chlorophyll traits based on canopy spectral reflectance and leaf extracts. *BMC Plant Biology*.

[12] Yang W., Guo Z., Huang C. (2014). Combining high-throughput phenotyping and genome-wide association studies to reveal natural genetic variation in rice. *Nature Communications*.

[13] Covarrubias-Pazaran G., Schlautman B., Diaz-Garcia L. (2018). Multivariate gblup improves accuracy of genomic selection for yield and fruit weight in biparental populations of vaccinium macrocarpon ait. *Frontiers in Plant Science*.

[14] Sun J., Rutkoski J. E., Poland J. A., Crossa J., Jannink J., Sorrells M. E. (2017). Multitrait, random regression, or simple repeatability model in high-throughput phenotyping data improve genomic prediction for wheat grain yield. *The Plant Genome*.

[15] Crain J., Mondal S., Rutkoski J., Singh R. P., Poland J. (2018). Combining high-throughput phenotyping and genomic information to increase prediction and selection accuracy in wheat breeding. *The Plant Genome*.

[16] Rutkoski J., Poland J., Mondal S. (2016). Canopy temperature and vegetation indices from high-throughput phenotyping improve accuracy of pedigree and genomic selection for grain yield in wheat. *G3: Genes, Genomes, Genetics*.

[17] Xavier A., Hall B., Hearst A. A., Cherkauer K. A., Rainey K. M. (2017). Genetic architecture of phenomic-enabled canopy coverage in glycine max. *Genetics*.

[18] Weber V., Araus J., Cairns J., Sanchez C., Melchinger A., Orsini E. (2012). Prediction of grain yield using reflectance spectra of canopy and leaves in maize plants grown under different water regimes. *Field Crops Research*.

[19] Montesinos-López O. A., Montesinos-López A., Crossa J. (2017). Predicting grain yield using canopy hyperspectral reflectance in wheat breeding data. *Plant Methods*.

[20] Ma B. L., Dwyer L. M., Costa C., Cober E. R., Morrison M. J. (2001). Early prediction of soybean yield from canopy reflectance measurements. *Agronomy Journal*.

[21] Christenson B. S., Schapaugh W. T., An N., Price K. P., Prasad V., Fritz A. K. (2016). Predicting soybean relative maturity and seed yield using canopy reflectance. *Crop Science*.

[22] Jia Y., Jannink J. (2012). Multiple-trait genomic selection methods increase genetic value prediction accuracy. *Genetics*.

[23] Bernardo R. (2002). *Breeding for Quantitative Traits in Plants*.

[24] Piepho H. P., Möhring J., Melchinger A. E., Büchse A. (2008). BLUP for phenotypic selection in plant breeding and variety testing. *Euphytica*.

[25] Dhondt S., Wuyts N., Inzé D. (2013). Cell to whole-plant phenotyping: the best is yet to come. *Trends in Plant Science*.

[26] Singh A. K., Ganapathysubramanian B., Sarkar S., Singh A. (2018). Deep learning for plant stress phenotyping: trends and future perspectives. *Trends in Plant Science*.

[27] Breiman L. (2001). Random forests. *Machine Learning*.

[28] Nagasubramanian K., Jones S., Sarkar S., Singh A. K., Singh A., Ganapathysubramanian B. (2018). Hyperspectral band selection using genetic algorithm and support vector machines for early identification of charcoal rot disease in soybean stems. *Plant Methods*.

[29] Ghosal S., Blystone D., Singh A. K., Ganapathysubramanian B., Singh A., Sarkar S. (2018). An explainable deep machine vision framework for plant stress phenotyping. *Proceedings of the National Acadamy of Sciences of the United States of America*.

[30] Thorp K., Wang G., Bronson K., Badaruddin M., Mon J. (2017). Hyperspectral data mining to identify relevant canopy spectral features for estimating durum wheat growth, nitrogen status, and grain yield. *Computers and Electronics in Agriculture*.

[31] Kaleita A. L., Steward B. L., Ewing R. P. (2006). Novel analysis of hyperspectral reflectance data for detecting onset of pollen shed in Maize. *Transactions of the ASABE*.

[32] Golberg D. E. (1989). *Genetic Algorithms in Search, Optimization, And Machine Learning*.

[33] Migicovsky Z. (2017). Patterns of genomic and phenomic diversity in wine and table grapes. *Horticulture Research*.

[34] Condorelli G. E. (2018). Comparative aerial and ground based high throughput phenotyping for the genetic dissection of ndvi as a proxy for drought adaptive traits in durum wheat. *Frontiers in Plant Science*.

[35] Wang C., Hu S., Gardner C., Lübberstedt T. (2017). Emerging avenues for utilization of exotic germplasm. *Trends in Plant Science*.

[36] Rebetzke G. J., Jimenez-Berni J., Fischer R. A., Deery D. M., Smith D. J. (2018). Review: High-throughput phenotyping to enhance the use of crop genetic resources. *Journal of Plant Sciences*.

[37] Oliveira M. F., Nelson R. L., Geraldi I. O., Cruz C. D., de Toledo J. F. (2010). Establishing a soybean germplasm core collection. *Field Crops Research*.

[38] Song Q. (2017). Genetic characterization of the soybean nested association mapping population. *The Plant Genome*.

[39] Fehr W. R., Caviness C. E., Burmood D. T., Pennington J. S. (1971). Stage of development descriptions for soybeans, glycine max (L.) Merrill1. *Crop Science*.

[40] Patrignani A., Ochsner T. E. (2015). Canopeo: A powerful new tool for measuring fractional green canopy cover. *Agronomy Journal*.

[41] Yang J., Zeng J., Goddard M. E., Wray N. R., Visscher P. M. (2017). Concepts, estimation and interpretation of SNP-based heritability. *Nature Genetics*.

[42] VanRaden P. M. (2008). Efficient methods to compute genomic predictions. *Journal of Dairy Science*.

[43] Wimmer V., Albrecht T., Auinger H., Schön C. (2012). Synbreed: a framework for the analysis of genomic prediction data using R. *Bioinformatics*.

[44] de los Campos G., Sorensen D., Gianola D. (2015). Genomic heritability: what is it?. *PLoS Genetics*.

[45] Covarrubias-Pazaran G. (2016). Genome-assisted prediction of quantitative traits using the R package sommer. *PLoS ONE*.

[46] Kuhn M. (2008). Building predictive models in R using the caret package. * Journal of Statistical Software *.

[47] Jarquín D., Lemes da Silva C., Gaynor R. C. (2017). Increasing genomic-enabled prediction accuracy by modeling genotype × environment interactions in kansas wheat. *The Plant Genome*.

[48] Mondal S., Rutkoski J. E., Velu G. (2016). Harnessing Diversity in wheat to enhance grain yield, climate resilience, disease and insect pest resistance and nutrition through conventional and modern breeding approaches. *Frontiers in Plant Science*.

[49] Muleta K. T., Bulli P., Zhang Z., Chen X., Pumphrey M. (2017). Unlocking diversity in germplasm collections via genomic selection: a case study based on quantitative adult plant resistance to stripe rust in spring wheat. *The Plant Genome*.

[50] Dinglasan E. G., Singh D., Shankar M. (2019). Discovering new alleles for yellow spot resistance in the Vavilov wheat collection. *Theoretical and Applied Genetics*.

[51] Bailey-Serres J., Fukao T., Ronald P., Ismail A., Heuer S., Mackill D. (2010). Submergence tolerant rice: sub1’s journey from landrace to modern cultivar. *Rice*.

[52] Meseka S., Fakorede M., Ajala S., Badu-Apraku B., Menkir A. (2013). Introgression of alleles from maize landraces to improve drought tolerance in an adapted germplasm. *Journal of Crop Improvement*.

[53] Kaler A. S., Ray J. D., Schapaugh W. T. (2018). Association mapping identifies loci for canopy temperature under drought in diverse soybean genotypes. *Euphytica*.

[54] Harris D. S., Schapaugh W. T., Kanemasu E. T. (1984). Genetic diversity in soybeans for leaf canopy temperature and the association of leaf canopy temperature and yield. *Crop Science*.

[55] Dwivedi S. L., Ceccarelli S., Blair M. W., Upadhyaya H. D., Are A. K., Ortiz R. (2016). Landrace germplasm for improving yield and abiotic stress adaptation. *Trends in Plant Science*.

[56] Mohammadi R., Haghparast R., Sadeghzadeh B., Ahmadi H., Solimani K., Amri A. (2014). Adaptation patterns and yield stability of durum wheat landraces to highland cold rainfed areas of Iran. *Crop Science*.

[57] DeLacy I. H., Basford K. E., Cooper M., Bull J. K., McLaren C. G. (1996). Analysis of multi-environment trials–an historical perspective. *Plant Adaptation and Crop Improvement*.

[58] Damesa T. M., Möhring J., Worku M., Piepho H. (2017). One step at a time: stage-wise analysis of a series of experiments. *Agronomy Journal*.

[59] Lorenz A. J. (2013). Resource allocation for maximizing prediction accuracy and genetic gain of genomic selection in plant breeding: A simulation experiment. *G3: Genes, Genomes, Genetics*.

[60] Vogelmann J. E., Rock B. N., Moss D. M. (1993). Red edge spectral measurements from sugar maple leaves. *International Journal of Remote Sensing*.

[61] Koester R. P., Nohl B. M., Diers B. W., Ainsworth E. A. (2016). Has photosynthetic capacity increased with 80 years of soybean breeding? An examination of historical soybean cultivars. *Plant, Cell & Environment*.

[62] Cozzolino D. (2017). The role of near-infrared sensors to measure water relationships in crops and plants. *Applied Spectroscopy Reviews*.

[63] Babar M. A., Reynolds M. P., van Ginkel M., Klatt A. R., Raun W. R., Stone M. L. (2006). Spectral reflectance indices as a potential indirect selection criteria for wheat yield under irrigation. *Crop Science*.

[64] El-Hendawy S. E., Hassan W. M., Al-Suhaibani N. A., Schmidhalter U. (2017). Spectral assessment of drought tolerance indices and grain yield in advanced spring wheat lines grown under full and limited water irrigation. *Agricultural Water Management*.

[65] Teal R. K., Tubana B., Girma K. (2006). In-season prediction of corn grain yield potential using normalized difference vegetation index. *Agronomy Journal*.

[66] Christenson B. S., Schapaugh W. T., An N., Price K. P., Fritz A. K. (2014). Characterizing changes in soybean spectral response curves with breeding advancements. *Crop Science*.

[67] Babar M. A., Reynolds M. P., van Ginkel M., Klatt A. R., Raun W. R., Stone M. L. (2006). Spectral reflectance to estimate genetic variation for in-season biomass, leaf chlorophyll, and canopy temperature in wheat. *Crop Science*.

[68] Gizaw S. A., Godoy J. G., Garland-Campbell K., Carter A. H. (2018). Using spectral reflectance indices as proxy phenotypes for genome-wide association studies of yield and yield stability in pacific northwest winter wheat. *Crop Science*.

[69] Akdemir D., Sanchez J. I., Jannink J. (2015). Optimization of genomic selection training populations with a genetic algorithm. *Genetics Selection Evolution*.

[70] Roger J. M., Bellon-Maurel V. (2016). Using genetic algorithms to select wavelengths in near-infrared spectra: application to sugar content prediction in cherries. *Applied Spectroscopy*.

[71] Heckmann D., Schlüter U., Weber A. P. (2017). Machine learning techniques for predicting crop photosynthetic capacity from leaf reflectance spectra. *Molecular Plant*.

[72] Shoeiby M., Robles-Kelly A., Timofte R. PIRM2018 challenge on spectral image super-resolution: methods and results.

[73] Arad B., Ben-Shahar O., Timofte R., Van Gool L., Zhang L., Yang M.-H. NTIRE 2018 challenge on spectral reconstruction from RGB images.

[74] Zhang Y., Qin Q., Ren H. (2018). Optimal hyperspectral characteristics determination for winter wheat yield prediction. *Remote Sensing*.

[75] Huang J., Wang X., Li X., Tian H., Pan Z. (2013). Remotely sensed rice yield prediction using multi-temporal ndvi data derived from NOAA's-AVHRR. *PLoS ONE*.

[76] Reynolds M., Langridge P. (2016). Physiological breeding. *Current Opinion in Plant Biology*.

[77] Mir R. R., Zaman-Allah M., Sreenivasulu N., Trethowan R., Varshney R. K. (2012). Integrated genomics, physiology and breeding approaches for improving drought tolerance in crops. *Theoretical and Applied Genetics*.

[78] Akdemir D., Beavis W., Fritsche-Neto R., Singh A. K., Isidro-Sánchez J. (2018). Multi-objective optimized genomic breeding strategies for sustainable food improvement. *Heredity*.

[79] Raun W. R., Solie J. B., Johnson G. V. (2001). In-season prediction of potential grain yield in winter wheat using canopy reflectance. *Agronomy Journal*.

[80] Prasad B., Carver B. F., Stone M. L., Babar M. A., Raun W. R., Klatt A. R. (2007). Genetic analysis of indirect selection for winter wheat grain yield using spectral reflectance indices. *Crop Science*.

[81] Gamon J. A., Serrano L., Surfus J. S. (1997). The photochemical reflectance index: an optical indicator of photosynthetic radiation use efficiency across species, functional types, and nutrient levels. *Oecologia*.

[82] Chappelle E. W., Kim M. S., McMurtrey J. E. (1992). Ratio analysis of reflectance spectra (RARS): an algorithm for the remote estimation of the concentrations of chlorophyll A, chlorophyll B, and carotenoids in soybean leaves. *Remote Sensing of Environment*.

[83] Serrano L., Peñuelas J., Ustin S. L. (2002). Remote sensing of nitrogen and lignin in Mediterranean vegetation from AVIRIS data: decomposing biochemical from structural signals. *Remote Sensing of Environment*.

[84] Wang L., Qu J. J. (2007). NMDI: A normalized multi-band drought index for monitoring soil and vegetation moisture with satellite remote sensing. *Geophysical Research Letters*.

[85] Roujean J.-L., Breon F.-M. (1995). Estimating PAR absorbed by vegetation from bidirectional reflectance measurements. *Remote Sensing of Environment*.

