# Supplementary material for: Development of Optimized Phenomic Predictors for Efficient Plant Breeding Decisions Using Phenomic-Assisted Selection in Soybean
Source: Plant Phenomics. 2019 Jul 28;2019:5809404. doi: 10.34133/2019/5809404 (PMC7706298; doi:10.34133/2019/5809404)
Supplement: Supplementary Materials — Table S1: description of accessions, country of origin, and genetic background included in this study. 292 accessions were selected from the USDA Soybean Core Collection from MGI-III. Table S2: description of testing environment locations, planting date, seed yield (SY) performance, and climactic summary statistics. Soybean accessions were phenotyped in these environments for use in downstream phenomic prediction. Table S3: description of vegetation indices (VI) computed from canopy hyperspectral reflectance. Observations consisted of two measurements recorded within 2 hours of solar noon and mean reflectance averaged. VIs were used alongside other phenomic information for in-season seed yield prediction [79–85]. Table S4: description of phenotypic traits and instruments used for phenotypic characterization of a diverse panel of soybean evaluated in six environments. Table S5: details of genetic algorithm (GA) procedure used for selection of hyperspectral wavebands for identifying the most informative wavebands to allow intelligent design of a miniaturized hyperspectral camera for deployment on high-throughput phenotyping platforms. Table S6: ANOVA results of fixed effects for mixed linear model where seed yield (SY) was the response variable. SY was collected from 292 genotypes grown in six environments across central Iowa and measured by combine harvest. Table S7: genetic correlation (rg) and SNP-based heritability of phenomic traits and seed yield and phenomic trait, respectively. Phenomic information was collected from 292 diverse soybean accessions grown in six environments across central Iowa and data collected during the growing seasons at two approximate growth stages. Table S8: phenomic traits feature importance computed from random forest model using two cross-validation scenarios while seed yield was used as the response variables. Phenomic traits were collected at two approximate growth stages and used to predict seed yield during the growing season to e [file 5809404.f1.docx]

**Table S1** Description of accessions, country of origin, and genetic background included in this study. 292 accessions were selected from the USDA Soybean Core Collection from MGI-III.

| **Accession** | **Origin** | **Diversity^a^** | **Accession** | **Origin** | **Diversity^a^** | **Accession** | **Origin** | **Diversity^a^** | **Accession** | **Origin** | **Diversity^a^** |
| --- | --- | --- | --- | --- | --- | --- | --- | --- | --- | --- | --- |
| 4J10534 | U.S. | E | PI437145B | Russia | PI | PI518751 | Yugoslavia | PI | PI603452 | China | PI |
| 5M20252 | U.S. | E | PI437169B | Russia | PI | PI518757 | Taiwan | PI | PI603470 | China | PI |
| CL0J09546 | U.S. | E | PI437340B | Russia | PI | PI532462A | China | PI | PI603546A | China | PI |
| CL0J17368 | U.S. | E | PI437356 | Russia | PI | PI538377 | China | PI | PI603560 | China | PI |
| HS63976 | U.S. | E | PI437377 | Russia | PI | PI538389 | Japan | PI | PI603594 | China | PI |
| IA3023 | U.S. | E | PI437399 | Russia | PI | PI538393 | China | PI | PI603596 | China | PI |
| LD015907 | U.S. | E | PI437427B | Russia | PI | PI538400 | China | PI | PI603655 | China | PI |
| LD024485 | U.S. | E | PI437462A | Russia | PI | PI54591 | China | PI | PI603660 | China | PI |
| LG032979 | U.S. | D | PI437477A | Russia | PI | PI54608_1 | China | PI | PI603662B | China | PI |
| LG044717 | U.S. | D | PI437581 | China | PI | PI548316 | China | PI | PI603674 | China | PI |
| LG054464 | U.S. | D | PI437585 | China | PI | PI548349 | North Korea | PI | PI603747 | China | PI |
| LG054832 | U.S. | D | PI437592 | China | PI | PI548373 | China | PI | PI603749 | China | PI |
| LG902550 | U.S. | D | PI437594A | China | PI | PI549021A | China | PI | PI603912 | North Korea | PI |
| LG921255 | U.S. | D | PI437651B | China | PI | PI549031 | China | PI | PI603915C | North Korea | PI |
| LG941128 | U.S. | D | PI437674 | China | PI | PI561227 | China | PI | PI612611 | North Korea | PI |
| LG941906 | U.S. | D | PI437682A | China | PI | PI561230 | China | PI | PI62202 | China | PI |
| LG977012 | U.S. | D | PI437690 | China | PI | PI561232 | China | PI | PI68788 | China | PI |
| LG981605 | U.S. | D | PI437715 | China | PI | PI561349 | China | PI | PI70463 | China | PI |
| Maverick | U.S. | E | PI437840A | China | PI | PI561370 | China | PI | PI72232 | China | PI |
| NE3001 | U.S. | E | PI437973 | China | PI | PI561377 | Japan | PI | PI79691_4 | China | PI |
| PI153280 | France | PI | PI438103 | China | PI | PI567161 | China | PI | PI80459 | Japan | PI |
| PI157421 | South Korea | PI | PI438133B | China | PI | PI567170A | China | PI | PI80461 | Japan | PI |
| PI167240 | Turkey | PI | PI438139 | China | PI | PI567170B | China | PI | PI80469 | Japan | PI |
| PI171450 | Japan | PI | PI438173 | China | PI | PI567229A | Russia | PI | PI80831 | China | PI |
| PI173994 | South Korea | PI | PI438194 | China | PI | PI567241 | China | PI | PI81044_2 | Japan | PI |
| PI181536 | Japan | PI | PI438259B | China | PI | PI567255A | China | PI | PI81667 | China | PI |
| PI181537 | Japan | PI | PI438292 | Japan | PI | PI567261B | China | PI | PI81763 | China | PI |
| PI189930 | France | PI | PI438312 | Algeria | PI | PI567262D | China | PI | PI81768 | China | PI |
| PI189969 | France | PI | PI438434 | Morocco | PI | PI567264A | China | PI | PI81773 | Japan | PI |
| PI200548 | Japan | PI | PI438503A | U.S. | PI | PI567266A | China | PI | PI82278 | South Korea | PI |
| PI227212 | Japan | PI | PI458052 | South Korea | PI | PI567267A | China | PI | PI84611 | South Korea | PI |
| PI232992 | Japan | PI | PI458061A | South Korea | PI | PI567275 | Japan | PI | PI84921 | North Korea | PI |
| PI253650A | China | PI | PI458110 | South Korea | PI | PI567351A | China | PI | PI84973 | Japan | PI |
| PI253658A | China | PI | PI458307A | South Korea | PI | PI567365 | China | PI | PI85009_1 | Japan | PI |
| PI253660B | China | PI | PI458506 | China | PI | PI567366B | China | PI | PI85356 | South Korea | PI |
| PI261466 | Japan | PI | PI458507 | China | PI | PI567538B | China | PI | PI86006 | Japan | PI |
| PI261474 | China | PI | PI458517 | China | PI | PI567583A | China | PI | PI86081 | Japan | PI |
| PI290134 | China | PI | PI458519A | China | PI | PI567595A | China | PI | PI86145 | Japan | PI |
| PI323586B | Portugal | PI | PI458520 | China | PI | PI567619 | China | PI | PI86449 | Japan | PI |
| PI339868E | South Korea | PI | PI458521 | China | PI | PI567644 | China | PI | PI86452 | Japan | PI |
| PI347552B | Russia | PI | PI458522 | China | PI | PI567729 | China | PI | PI87600_1 | North Korea | PI |
| PI361080 | Russia | PI | PI464877 | China | PI | PI567774B | China | PI | PI87618 | North Korea | PI |
| PI361101 | Korea | PI | PI464880 | China | PI | PI574478B | China | PI | PI87631_1 | Japan | PI |
| PI379559D | Japan | PI | PI467310 | China | PI | PI574480B | China | PI | PI87634 | Japan | PI |
| PI379561 | Japan | PI | PI467312 | China | PI | PI574486 | China | PI | PI88289 | China | PI |
| PI391577 | China | PI | PI467327 | China | PI | PI578360 | China | PI | PI88292 | China | PI |
| PI391586 | China | PI | PI467332 | China | PI | PI578363 | China | PI | PI88305 | China | PI |
| PI398813 | South Korea | PI | PI468384 | China | PI | PI578364 | China | PI | PI88306 | China | PI |
| PI398881 | South Korea | PI | PI468385 | China | PI | PI578366 | China | PI | PI88788 | China | PI |
| PI404160B | Georgia | PI | PI470223 | China | PI | PI578367 | China | PI | PI89003_1 | China | PI |
| PI404166 | China | PI | PI470227B | China | PI | PI578376 | China | PI | PI89008 | China | PI |
| PI404169B | China | PI | PI475810 | China | PI | PI578380A | China | PI | PI89130 | North Korea | PI |
| PI404188A | China | PI | PI475818 | China | PI | PI578416 | China | PI | PI89134 | North Korea | PI |
| PI407653 | China | PI | PI475820 | China | PI | PI578439 | Vietnam | PI | PI89152 | North Korea | PI |
| PI407656 | China | PI | PI475822B | China | PI | PI578473A | China | PI | PI89153 | North Korea | PI |
| PI407746 | China | PI | PI476344 | Uzbekistan | PI | PI578499A | China | PI | PI89154 | North Korea | PI |
| PI407810 | South Korea | PI | PI476911 | Vietnam | PI | PI578499B | China | PI | PI89773 | China | PI |
| PI416773 | Japan | PI | PI479711 | China | PI | PI588008A | China | PI | PI91091 | China | PI |
| PI416835 | Japan | PI | PI479713 | China | PI | PI592907C | Russia | PI | PI91102 | China | PI |
| PI416868A | Japan | PI | PI479718B | China | PI | PI592910 | Russia | PI | PI91120_3 | China | PI |
| PI417054 | Japan | PI | PI479729 | China | PI | PI592911B | Russia | PI | PI91162 | China | PI |
| PI417138 | Japan | PI | PI479740 | China | PI | PI593970 | Japan | PI | PI91341 | China | PI |
| PI417167 | Japan | PI | PI506527 | Japan | PI | PI594156 | Japan | PI | PI91349 | China | PI |
| PI417198 | Japan | PI | PI506529 | Japan | PI | PI594394 | China | PI | PI92603 | China | PI |
| PI417297 | Japan | PI | PI506800B | Japan | PI | PI594457A | China | PI | PI92611 | China | PI |
| PI417559 | Poland | PI | PI506887 | Japan | PI | PI594471A | China | PI | PI92683 | China | PI |
| PI427136 | South Korea | PI | PI507147 | Japan | PI | PI597482 | South Korea | PI | PI96162 | North Korea | PI |
| PI430596 | China | PI | PI507171 | Japan | PI | PI603367 | China | PI | PI96199 | China | PI |
| PI430597 | China | PI | PI507267 | Japan | PI | PI603412B | China | PI | PI96322 | North Korea | PI |
| PI430619 | China | PI | PI507487 | Japan | PI | PI603422B | China | PI | PI96786_1 | North Korea | PI |
| PI437121B | Russia | PI | PI507491 | Japan | PI | PI603428D | China | PI | Prohio | U.S. | E |
| PI437122 | Russia | PI | PI507681B | Uzbekistan | PI | PI603438E | China | PI | Skylla | U.S. | E |
| PI437124 | Georgia | PI | PI518283 | Taiwan | PI | PI603442 | China | PI | U03100612 | U.S. | E |

^a^ (E) Elite, (D) Diverse

**Table S2** Description of testing environment locations, planting date, seed yield (SY) performance, and climactic summary statistics. Soybean accessions were phenotyped in these environments for use in downstream phenomic prediction.

|  |  |  |  |  | **Seed Yield** | |  | **Avg Temp^b^** | |  |  |  |
| --- | --- | --- | --- | --- | --- | --- | --- | --- | --- | --- | --- | --- |
| **Year** | **Environment** | **GPS Coordinates** | **Planting Date** |  | **Mean^a^** | **SD** |  | **Low** | **High** |  | **Precip^c^** | **Solar^d^** |
| 2016 | Env1 | 42°01'02.5"N 93°46'19.6"W | 5/21/2016 |  | 2637 | 770.6 |  | 15.5 | 27 |  | 629.9 | 3031 |
|  | Env2 | 41°19'44.0"N 95°10'58.3"W | 6/09/2016 |  | 2009 | 750 |  | 15.2 | 27 |  | 649 | 2834 |
| 2017 | Env3 | 42°01'17.5"N 93°46'04.2"W | 6/15/2017 |  | 1960 | 909.7 |  | 14.6 | 27.2 |  | 374.4 | 3128 |
|  | Env4 | 42°03'35.9"N 95°50'17.7"W | 6/25/2017 |  | 2130 | 909.9 |  | 14.6 | 27 |  | 519.4 | 3291 |
|  | Env5 | 41°19'51.8"N 95°10'58.9"W | 6/26/2017 |  | 2510 | 955.9 |  | 14.2 | 26.6 |  | 563.3 | 3256 |
|  | Env6 | 42°00'35.8"N 93°46'41.5"W | 6/29/2017 |  | 1522 | 861.4 |  | 14.6 | 27.2 |  | 374.4 | 3128 |

Note: Env3 and Env6 shared the same climactic information due to their relative proximity to each other

^a^ Mean seed yield (kg ha^-1^)

^b^ Average low and high temperature measured as degrees Celsius during the growing season (May 1 – September 31)

^c^ Cumulative precipitation (mm) during the growing season (May 1 – September 31)

^d^ Cumulative solar radiation (MJ m^-2^) during the growing season (May 1 – September 31)

**Table S3** Description of vegetation indices (VI) computed from canopy hyperspectral reflectance. Observations consisted of two measurements recorded within 2 hours of solar noon and mean reflectance averaged. VI’s were used alongside other phenomic information for in-season seed yield prediction.

| **Vegetation Index** | **Formula^a^** | **Associated Trait** | **Reference** |
| --- | --- | --- | --- |
| Normalized Vegetation Index (NDVI) | R_780_-R_680_/R_780_+R_680_ | Green biomass, chlorophyll content | [79] |
| Normalized Water Index (NWI) | R_970_-R_900_/R_970_+R_900_ | Plant water status | [80] |
| Photochemical Reflectance Index (PRI) | R_531_-R_570_/R_531_+R_570_ | Photosynthetic radiation use efficiency | [81] |
| Ratio Analysis of Reflectance Spectra Chlorophyll a (RARSa) | R_675_ /R_700_ | Chlorophyll a content | [82] |
| Ratio Analysis of Reflectance Spectra Chlorophyll b (RARSb) | R_675_ /(R_650_*R_700_) | Chlorophyll b content | [82] |
| Ratio Analysis of Reflectance Spectra Chlorophyll c (RARSb) | R_760_/R_500_ | Carotenoid content | [82] |
| Vogelmann Red Edge Index 2 (VREI2) | R_734_-R_747_/R_715_+R_726_ | Total leaf chlorophyll content | [60] |
| Normalized Lignin Index (NDLI) | [log(1/R_1754_)-log(1/R_1680_)]/ [log(1/R_1754_)+log(1/R_1680_)] | Bulk canopy lignin content | [83] |
| Normalized Multi-band Drought Index (NMDI) | [R_860_-(R_1640_ – R_2130_)]/[R_860_+(R_1640_– R_2130_)] | Vegetation moisture status | [84] |
| Red Normalized Difference Vegetation Index (RDVI) | R_800_-R_670_/R_800_+R_670_ | Soil background adjusted biomass | [85] |

^a^ R indicates wavelength of spectral reflectance

**Table S4** Description of phenotypic traits and instruments used for phenotypic characterization of a diverse panel of soybean evaluated in six environments.

|  |  |  |  | **Environments Collected** | |
| --- | --- | --- | --- | --- | --- |
| **Trait** | **Instrument** | **Associated Trait** |  | **R1-R2 (S1)** | **R3-R4 (S2)** |
| Canopy Temperature (CT) | FLIR Vue Pro R 640 9mm^a^ | Plant water content |  | - | 3,4,5,6 |
| Spectral Reflectance (VI, R) | ASD Field Spec 4 Hi-Res^b^ | Vegetation indices/Raw Reflectance |  | All | All |
| Canopy Area (CA) | Canon T3i RGB Camera 18-55mm^c^ | Fractional Green Canopy Cover |  | 3,4,5,6 | 3,4,5,6 |

^a^ FLIR Systems, Wilsonville, OR, USA

^b^ Malvern Panalytical Inc., Westborough, MA, USA

^c^ Canon Inc., Tokyo,

**Table S5** Details of Genetic Algorithm (GA) procedure used for selection of hyperspectral wavebands for identifying the most informative wavebands to allow intelligent design of a miniaturized hyperspectral camera for deployment on high throughput phenotyping platforms.

| **Parameters** |  |
| --- | --- |
| Number of GA iterations | 10 |
| Population | 300 |
| Maximum Number of generations | 100 |
| Crossover Probability | 0.8 |
| Elite Count | 2 |
| Mutation Probability | 0.2 |
| Selection | Binary selection tournament |
| Crossover | Laplace crossover |
| Mutation | Power mutation |
| Stopping Criteria | Average change in best fitness value is less than 10^-6 for 50 generations or number of generations=100 |

The cost function of the GA was to maximize the R2 value of the random forest regression model.

**Table S6** ANOVA results of fixed effects for mixed linear model where seed yield (SY) was the response variable. SY was collected from 292 genotypes grown in six environments across central Iowa and measured by combine harvest.

| **Source of Variation** | **F value and significance level** | **df** |
| --- | --- | --- |
| Environment (e) | 617.8^*^ | 5 |
| Genotype (g) | 45.2^*^ | 291 |
| Genotype x Environment (ge) | 1.8^*^ | 1455 |

*Significance at the 0.01 level

**Table S7** Genetic correlation ($r_{g}$) and SNP-based heritability of phenomic traits and seed yield and phenomic trait, respectively. Phenomic information was collected from 292 diverse soybean accessions grown in six environments across central Iowa and data collected during the growing seasons at two approximate growth stages.

|  | **Growth Stage 1 (S1)** | |  | **Growth Stage 2 (S2)** | |
| --- | --- | --- | --- | --- | --- |
| **Trait** | **r_g_ (±SE)** | $\mathbf{h}_{\mathbf{SNP}}^{\mathbf{2}}$ **(±SE)** |  | **r_g_ (±SE)** | $\mathbf{h}_{\mathbf{SNP}}^{\mathbf{2}}$ **(±SE)** |
| CA | 0.33 (0.09) | 0.5 (0.09) |  | 0.25 (0.11) | 0.29 (0.08) |
| CT | - | - |  | -0.44 (0.11) | 0.38 (0.09) |
| VI_NDVI | 0.38 (0.11) | 0.29 (0.08) |  | 0.33 (0.07) | 0.73 (0.07) |
| VI_NWI | -0.58 (0.11) | 0.29 (0.08) |  | -0.59 (0.08) | 0.46 (0.09) |
| VI_PRI | 0.48 (0.10) | 0.37 (0.09) |  | 0.51 (0.08) | 0.54 (0.09) |
| VI_RARSa | 0.03 (0.13) | 0.25 (0.08) |  | 0.12 (0.10) | 0.58 (0.08) |
| VI_RARSb | 0.59 (0.13) | 0.31 (0.08) |  | 0.50 (0.09) | 0.59 (0.08) |
| VI_RARSc | 0.60 (0.12) | 0.25 (0.08) |  | 0.43 (0.09) | 0.52 (0.08) |
| VI_VREI2 | -0.77 (0.07) | 0.51 (0.09) |  | -0.75 (0.06) | 0.65 (0.08) |
| VI_NDLI | 0.45 (0.13) | 0.25 (0.08) |  | 0.30 (0.10) | 0.40 (0.09) |
| VI_NMDI | 0.03 (0.22) | 0.07 (0.04) |  | 0.59 (0.09) | 0.45 (0.09) |
| VI_RDVI | 0.30 (0.09) | 0.43 (0.09) |  | 0.34 (0.09) | 0.52 (0.09) |

**Table S8** Phenomic traits feature importance computed from random forest model using two cross-validation scenarios while seed yield was used as the response variables. Phenomic traits were collected at two approximate growth stages and used to predict seed yield during the growing season to enable in-season selection. Feature importance was used to select the most informative vegetation indice and to identify other useful predictors of seed yield.

| **Predictor** | **Method** | **CV1** | **CV2** |
| --- | --- | --- | --- |
| S1_VI_NDLI | 1 | 3.3 | 5.2 |
| S1_VI_NDVI | 1 | 13.3 | 14.3 |
| S1_VI_NMDI | 1 | 3.9 | 2.2 |
| S1_VI_NWI | 1 | 37.1 | 31.7 |
| S1_VI_PRI | 1 | 30.1 | 36.2 |
| S1_VI_RARSa | 1 | 21.5 | 22.4 |
| S1_VI_RARSb | 1 | 12.1 | 12.7 |
| S1_VI_RARSc | 1 | 28.6 | 22.4 |
| S1_VI_RDVI | 1 | 11.6 | 13.7 |
| S1_VI_VREI2 | 1 | 54.1 | 53.1 |
| S2_VI_NDLI | 1 | 29.8 | 27.1 |
| S2_VI_NDVI | 1 | 13.1 | 18.9 |
| S2_VI_NMDI | 1 | 23.3 | 22.2 |
| S2_VI_NWI | 1 | 23.9 | 24.1 |
| S2_VI_PRI | 1 | 39.8 | 48.9 |
| S2_VI_RARSa | 1 | 39.3 | 42.6 |
| S2_VI_RARSb | 1 | 27.2 | 31.4 |
| S2_VI_RARSc | 1 | 6.9 | 9.9 |
| S2_VI_RDVI | 1 | 35.2 | 39.5 |
| S2_VI_VREI2 | 1 | 100 | 99.8 |
| S1_CA | 1 | 100 | 98.2 |
| S2_CA | 1 | 15.6 | 30 |
| S2_CT | 1 | 22.2 | 14.9 |
| S1_VI_NDLI | 2 | 19.3 | 18.2 |
| S1_VI_NDVI | 2 | 40.4 | 30.1 |
| S1_VI_NMDI | 2 | 0 | 0.1 |
| S1_VI_NWI | 2 | 57.1 | 48.6 |
| S1_VI_PRI | 2 | 43.9 | 44.1 |
| S1_VI_RARSa | 2 | 31.7 | 34.2 |
| S1_VI_RARSb | 2 | 25.5 | 28.9 |
| S1_VI_RARSc | 2 | 18.6 | 22.4 |
| S1_VI_RDVI | 2 | 41 | 45.1 |
| S1_VI_VREI2 | 2 | 48.1 | 53.8 |
| S2_VI_NDLI | 2 | 31.7 | 33.7 |
| S2_VI_NDVI | 2 | 37.3 | 45.7 |
| S2_VI_NMDI | 2 | 36.6 | 39.7 |
| S2_VI_NWI | 2 | 53.3 | 60.5 |
| S2_VI_PRI | 2 | 85.3 | 86.8 |
| S2_VI_RARSa | 2 | 50.8 | 51.1 |
| S2_VI_RARSb | 2 | 61.5 | 61.9 |
| S2_VI_RARSc | 2 | 27.7 | 31.4 |
| S2_VI_RDVI | 2 | 60.6 | 64.5 |
| S2_VI_VREI2 | 2 | 99.4 | 99.3 |
| S1_CA | 2 | 98.6 | 95.1 |
| S2_CA | 2 | 5.4 | 15.9 |
| S2_CT | 2 | 38.1 | 37 |

**Table S9** Spearman rank correlation obtained after random forest model prediction (seed yield = dependent variable) performance of predictors trained with remotely sensed phenomic traits (canopy traits, waveband, vegetation indices and combination) in 292 soybean genotypes grown at six environments and data collected at two growth stages in each environment. Tabular data corresponding to Figure 4.

| **CV Method** | **Method** | **Predictors** | **Spearman Rank (±SD)** |
| --- | --- | --- | --- |
| CV1 | Method 1 | Canopy | 0.53 (0.03) |
|  |  | Canopy + VI | 0.76 (0.02) |
|  |  | VI | 0.75 (0.03) |
|  |  | Wave | 0.71 (0.03) |
|  | Method 2 | Canopy | 0.30 (0.03) |
|  |  | Canopy + VI | 0.68 (0.03) |
|  |  | VI | 0.67 (0.02) |
|  |  | Wave | 0.45 (0.03) |
| CV2 | Method 1 | Canopy | 0.29 (0.15) |
|  |  | Canopy + VI | 0.66 (0.09) |
|  |  | VI | 0.67 (0.09) |
|  |  | Wave | 0.44 (0.12) |
|  | Method 2 | Canopy | 0.30 (0.19) |
|  |  | Canopy + VI | 0.62 (0.08) |
|  |  | VI | 0.61 (0.08) |
|  |  | Wave | 0.37 (0.15) |

**Table S10** Spearman rank correlation and classification metrics of random forest model test prediction using only optimized wavebands and selected canopy traits. Applicability of using phenomic prediction in plant breeding operations was tested using four training/testing splits (80/20, 60/40, 40/60, 20/80) and performance metrics were computed for each split. Seed yield and phenomic predictor trait data were collected from 292 genotypes grown in six environments and data collected at two growth stages in each environment. Tabular data corresponding to Figure 5.

|  |  |  | **% Training Data** | | | |
| --- | --- | --- | --- | --- | --- | --- |
| **CV Method** | **Predictors** | **Metric** | **80** | **60** | **40** | **20** |
| **CV1** | **Wavebands** | Balanced Accuracy | 0.79 | 0.78 | 0.77 | 0.74 |
|  |  | F Score | 0.66 | 0.65 | 0.63 | 0.59 |
|  |  | Spearman Rank | 0.74 | 0.72 | 0.71 | 0.67 |
|  |  | Specificity | 0.91 | 0.91 | 0.91 | 0.90 |
|  |  |  |  |  |  |  |
|  | **Wavebands + VI + Canopy** | Balanced Accuracy | 0.85 | 0.83 | 0.83 | 0.82 |
|  |  | F Score | 0.75 | 0.73 | 0.72 | 0.71 |
|  |  | Spearman Rank | 0.79 | 0.78 | 0.77 | 0.76 |
|  |  | Specificity | 0.94 | 0.93 | 0.93 | 0.93 |
|  |  |  |  |  |  |  |
| **CV2** | **Wavebands** | Balanced Accuracy | 0.59 | 0.60 | 0.60 | 0.53 |
|  |  | F Score | 0.35 | 0.35 | 0.35 | 0.25 |
|  |  | Spearman Rank | 0.33 | 0.35 | 0.38 | 0.33 |
|  |  | Specificity | 0.84 | 0.84 | 0.84 | 0.81 |
|  |  |  |  |  |  |  |
|  | **Wavebands + VI + Canopy** | Balanced Accuracy | 0.77 | 0.73 | 0.77 | 0.77 |
|  |  | F Score | 0.64 | 0.58 | 0.63 | 0.63 |
|  |  | Spearman Rank | 0.61 | 0.61 | 0.62 | 0.62 |
|  |  | Specificity | 0.91 | 0.89 | 0.91 | 0.91 |
